# Suitability, Efficacy, and Safety of Vernakalant for New Onset Atrial Fibrillation in Critically Ill Patients

**DOI:** 10.1155/2014/826286

**Published:** 2014-05-12

**Authors:** Alain Rudiger, Alexander Breitenstein, Mattia Arrigo, Sacha P. Salzberg, Dominique Bettex

**Affiliations:** ^1^Cardiosurgical Intensive Care Unit, Institute of Anesthesiology, University Hospital Zurich, Raemistrasse 100, 8091 Zurich, Switzerland; ^2^Clinic for Cardiology, University Heart Center, University Hospital Zurich, Raemistrasse 100, 8091 Zurich, Switzerland; ^3^Clinic for Cardiac and Vascular Surgery, University Hospital Zurich, Raemistrasse 100, 8091 Zurich, Switzerland

## Abstract

*Objectives*. This study investigates the suitability, safety, and efficacy of vernakalant in critically ill patients with new onset atrial fibrillation (AF) after cardiac surgery. *Methods*. Patients were screened for inclusion and exclusion criteria according to the manufacturers' recommendations. Included patients were treated with 3 mg/kg of vernakalant over 10 min and, if unsuccessful, a second dose of 2 mg/kg. Blood pressure was measured continuously for 2 hours after treatment. *Results*. Of the 191 patients screened, 159 (83%) were excluded, most importantly due to hemodynamic instability (59%). Vernakalant was administered to 32 (17% of the screened) patients. Within 6 hours, 17 (53%) patients converted to sinus rhythm. Blood pressure did not decrease significantly 10, 30, 60, and 120 minutes after the vernakalant infusion. However, 11 patients (34%) experienced a transient decrease in mean arterial blood pressure <60 mmHg. Other adverse events included nausea (*n* = 1) and bradycardia (*n* = 2). *Conclusions*. Applying the strict inclusion and exclusion criteria provided by the manufacturer, only a minority of postoperative ICU patients with new onset AF qualified for vernakalant. Half of the treated patients converted to sinus rhythm. The drug was well tolerated, but close heart rate and blood pressure monitoring remains recommended.

## 1. Background

Atrial fibrillation (AF) remains a clinical challenge for physicians caring for critically ill patients [1]. It is frequent in patients hospitalized in the intensive care unit (ICU) and particularly common in patients after cardiac surgery [2, 3]. Up to a third of patients undergoing coronary artery bypass grafting (CABG) and/or valve surgery develop postoperative AF [4, 5]. It is often poorly tolerated, particularly in the first days postoperatively or in cases of diastolic dysfunction.

Intravenous amiodarone is commonly used in the ICU setting, even though the conversion rate into sinus rhythm is moderate [6, 7]. A wide range of cardiac (e.g., negative inotropic, QT prolongation) and noncardiac adverse effects (e.g., thyroid disorders, liver and lung toxicity, neuropathy, and photosensitivity) as well as a very long half-life can cause problems in treated patients. Other antiarrhythmic drugs such as propafenone, flecainide, or ibutilide can rarely be used in ICU patients with AF, because they are contraindicated in patients with structural heart disease due to a high risk of proarrhythmic side effects [8, 9]. Hence, rapidly acting, efficacious, and well-tolerated drugs for the treatment of AF are urgently needed.

Vernakalant is a novel antiarrhythmic agent with an atrial-specific mechanism of action [10]. It is a multichannel blocker which inhibits the potassium currents *I*_kur_ and *I*_KACh_, resulting in a prolongation of the action potential repolarization. Additionally, vernakalant exerts an inhibitory action on sodium channels [11]. The atrium-specific effect was demonstrated in an electrophysiology study in 19 patients, in which the intravenous application of vernakalant significantly prolonged the atrial, but not the ventricular, refractory period [12]. The drug is metabolized by cytochrome P2D6. The elimination half-life in fast and slow metabolizers is 3 and 5.5 hours, respectively. No dose adjustments are needed in patients with advanced age, renal and liver dysfunction.

Case reports indicate that vernakalant might be an interesting treatment option in critically ill patients [13]. However, systematic reports on the usefulness of this substance in this particular population are lacking. Therefore, the current study was designed to answer the following questions.

1. How many ICU patients with new onset AF after cardiac surgery qualify for vernakalant according to inclusion and exclusion criteria provided by the official drug information (suitability)?
2. What is the conversion rate to sinus rhythm after administration of vernakalant in critically ill patients (efficacy)?
3. What hemodynamic and adverse effects of vernakalant occur in responders and nonresponders (safety)?

## 2. Methods

### 2.1. Patients

The study was performed in the Cardiosurgical ICU at the University Hospital Zurich, Switzerland. In the year 2012, 1,044 patients were admitted to this 12-bed unit, the majority of them after cardiac or major vascular surgery. Median SAPS and ICU length of stay of all admitted patients were 27 and 3.5 days, respectively. Patients were included into this study if they were ≥18 years old and suffered from new onset (<7 days) AF. Electrocardiogram (ECG), blood pressure, and oxygen saturation were monitored continuously in all patients. Patients did not qualify for vernakalant and were therefore excluded from the survey, if they fulfilled one or more of the following criteria: cardiovascular instability (noradrenaline dose ≥0.1 mcg/kg/min); need for inotropes; atrial flutter; myocardial infarction (ST elevation or non-ST elevation) within 30 days; QT_(not  corrected)_ interval >440 ms; intravenous administration of antiarrhythmic drugs of classes I or III (amiodarone) 4 hours prior to the planned vernakalant infusion; liver cirrhosis Child C; pregnancy, lactation; allergy against vernakalant. In order to reduce the risk of thromboembolic complications, vernakalant was only administered, if (a) AF persisted for less than 48 hour, (b) a surgical left atrial appendage occlusion had been performed during cardiac surgery, or (c) a thrombus in the atrium or left atrial appendage was excluded by transesophageal echocardiography. Potassium serum levels were kept between 4.5 and 5.5 mmol/L. Magnesium was substituted prior to the study drug infusion if the serum level was <1.0 mmol/L.

### 2.2. Vernakalant

Vernakalant (Brinavess) was administered via infusion according to the manufacturer's recommendations at a dose of 3 mg/kg over 10 minutes. If no conversion occurred 15 minutes after the end of the first infusion, a second dose of 2 mg/kg was administered as an infusion over 10 minutes. In patients weighing ≥100 kg, first and second doses of 300 mg and 200 mg were used (500 mg maximum). Responders were defined as patients who converted to sinus rhythm within 6 hours after the vernakalant administration.

### 2.3. Monitoring

Patient characteristics and laboratory values were recorded from the patients' charts. No extra blood was taken or analyzed for this study. Heart rhythm and rate as well as blood pressure were monitored continuously for at least 2 hours after the vernakalant infusion and recorded 10, 30, 60, and 120 minutes after the first infusion. Adverse events were recorded at any time point, but symptoms such as nausea or dysgeusia were not particularly asked for.

### 2.4. Statistics

All data were collected from the patient's charts and entered into a database. Median (range) or percentages were calculated for the overall sample and subgroups. Comparisons between groups were made with the use of the Mann-Whitney *U* test or the Fisher exact test, as appropriate. Repeated measures within groups were compared with a Wilcoxon signed rank sum test. The null hypothesis was rejected with a two-sided *P* value of <0.05. All analyses were performed with the use of SPSS for Mac OS X.

### 2.5. Ethical Considerations

Vernakalant has been licensed in Switzerland and the drug was used according to the manufacturer's recommendation. As the study was designed as a survey (field report), no informed consent was required for this type of study. The study protocol was approved by the Clinical Trial Center at the University Zurich and the local ethical committee (KEK-ZH-Nr. 2011-0396).

## 3. Results

### 3.1. Suitability

A total of 191 patients with AF were screened (132 men, 59 women). Of these patients, 159 (83%) were excluded due to hemodynamic instability (*n* = 113; 59%), self-limiting AF (*n* = 19; 9.9%), chronic AF (*n* = 13; 6.8%), prior intravenous amiodarone treatment (*n* = 11; 5.8%), myocardial infarction (*n* = 7; 3.7%), and/or long QT interval (*n* = 5; 2.6%). Vernakalant was administered to 32 (17% of the screened) patients. In these patients, AF occurred on average on the 4th (1st–16th) postoperative day. Baseline characteristics and laboratory parameters prior to vernakalant treatment are given in Tables 1 and 2. Five patients (16%) had a left-ventricular ejection fraction ≤45%.

### 3.2. Efficacy

Six patients (21%) converted directly after the first dose of vernakalant, but 26 (79%) patients did not and required a second dose. Within 6 hours, 17 (53%) patients converted to sinus rhythm (responders) and 15 (47%) did not (nonresponders). The time to conversion was 30 (4–355) minutes in responders. AF was normocard in 5 (33%) nonresponders and in 2 (12%) responders (*P* = 0.21). Responders had slightly but significantly lower magnesium levels at baseline than nonresponders (Table 2). Intravenous magnesium sulfate (22 mval) was administered to 5 (33%) nonresponders and 9 (53%) responders (*P* = 0.31) prior to the vernakalant infusion. Of the responders, 5 (29%) had a relapse of AF during the 6-hour observation period. Outcome parameters are given in Table 4.

### 3.3. Safety

**Figure 1:**
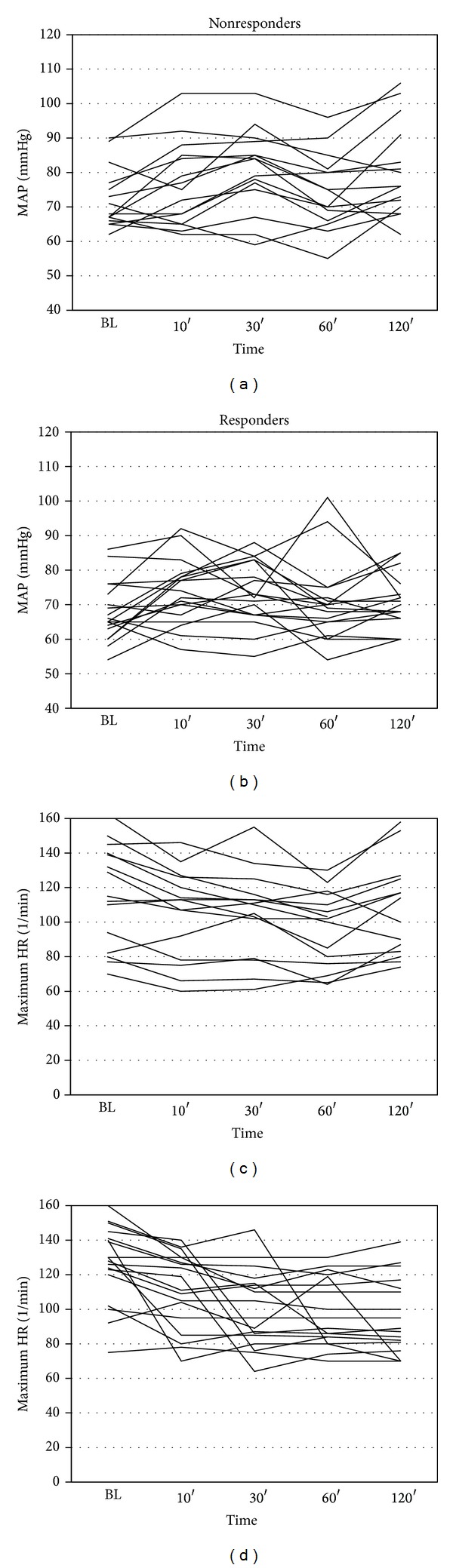
The lines show individual changes of mean arterial pressure (MAP) and heart rate (HR) of nonresponders and responders during the 2 h observation period.

**Figure 2:**
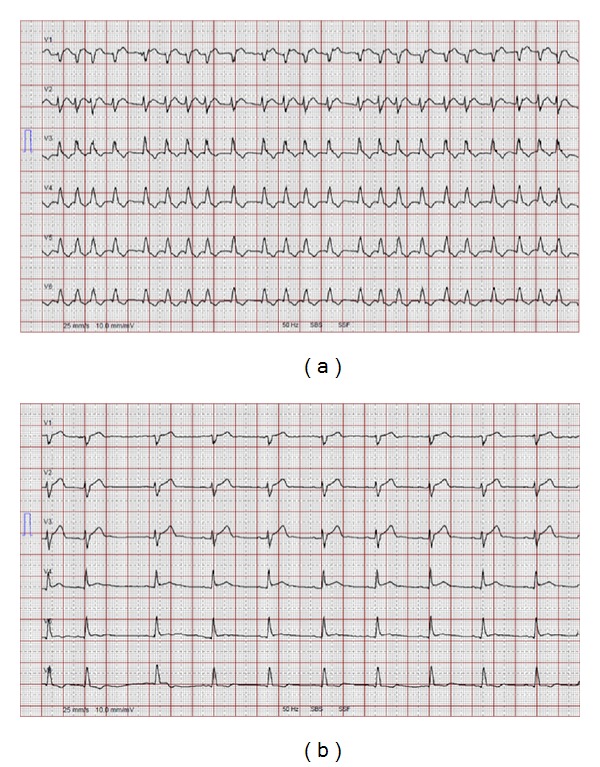
(a) The ECG of this 61-year-old patient 3 days after aortocoronary bypass grafting shows atrial fibrillation (HR 129–136/min) and a left bundle branch block. (b) After 2 doses of vernakalant, the patient converted into sinus rhythm (HR 57/min). Bradycardia decreased SvO_2_ to 49% and required temporary atrial pacing via the existing epicardial leads.

Hemodynamic changes are illustrated in Figure 1 and Table 3. A drop in heart rate <60/min was documented in 2 patients (6.3%), of which 1 episode led to a transient drop in SvO_2_ (Figure 2). A decrease in mean arterial BP <60 mmHg occurred in 3 (27%) of the nonresponders and 8 (47%) of the responders (*P* = 0.15). Four patients (12.5%; 2 responders and 2 nonresponders) required therefore either noradrenaline boli or an increase of the noradrenaline infusion rate >0.1 mcg/kg/min. One patient reported nausea, but no patient complained about dysgeusia. No patient developed ventricular tachycardia or torsades de pointes.

## 4. Discussion

In this postmarketing survey, we assessed the suitability, efficacy, and safety of vernakalant in ICU patients with new onset AF after cardiac surgery. To our knowledge, this is the first report focusing on the effects of vernakalant in critically ill patients with a high prevalence of structural heart disease.

### 4.1. Suitability

Strictly applying inclusion and exclusion criteria recommended by the manufacturer, the majority of screened patients could not be treated with vernakalant. The most important exclusion criterion was hemodynamic instability, which was defined as a noradrenaline requirement ≥0.1 mcg/kg/min, the need of inotropes, or the use of a mechanical cardiac assist device. Other relevant exclusion criteria were previous or ongoing treatment with intravenous amiodarone, a history of recent myocardial infarction, and QT prolongation >440 ms (not corrected). At the end, 32 critically ill patients with new onset AF were treated with vernakalant, which is to date the largest series of vernakalant-treated ICU patients. The high severity of illness in this population is reflected by the facts that median SAPS was 37, 44% were on mechanical ventilation, 34% required low-dose vasopressors, and 22% of the patients depended on dialysis. Of note, the majority of patients suffered from systemic inflammation with median values of white blood cell count and C-reactive protein level of 12 × 10^9^/L and 123 mg/L, respectively.

### 4.2. Efficacy

When vernakalant was administered at a first dose of 3 mg/kg over 10 minutes with a second dose of 2 mg/kg if necessary, more than half of the patients converted from AF to sinus rhythm. This conversion rate is similar to published results from previous trials performed in non-ICU patients. The CRAFT trial (conversion of rapid atrial fibrillation trial), a phase II multicenter clinical trial, randomized 56 patients to placebo (*n* = 20) and two different dosages of vernakalant (*n* = 18 for each group) [14]. High-dose treatment with vernakalant (2 mg/kg over 10 minutes followed by 3 mg/kg if AF was still present after the first dose) converted AF to sinus rhythm in 61% of treated patients versus 5% of patients in the control group. The ACT (atrial arrhythmia conversion) trials I–IV [15–18] were phase III studies demonstrating efficacy and safety of vernakalant in hemodynamic stable patients with new onset AF. The drug was generally well tolerated with a conversion rate consistently around 50%. Kowey et al. compared vernakalant and placebo in noncritically ill patients with AF after cardiac surgery, and the conversion rates within 90 minutes of dosing were 47% and 14%, respectively [17]. The AVRO study (phase III superiority study of vernakalant versus amiodarone in subjects with recent onset atrial fibrillation) demonstrated that vernakalant was more effective than amiodarone for the rapid conversion of AF to sinus rhythm (51.7% versus 5.7% at 90 minutes after start of treatment) [19]. Recently, Torp-Pedersen and coworkers demonstrated that the conversion rate of vernakalant was independent of a history of ischemic heart disease [20].

However, some of our patients had a relapse to AF, leading to a final conversion rate of 33% beyond 6 hours. Interestingly, this conversion rate is still better than that after electrical conversion in critically ill patients. Mayr et al. described success rates immediately, 24 hours, and 48 hours after electrical conversion of 35%, 24%, and 16%, respectively [21]. Hence, the conversion rates of electrical cardioversion are much lower in critically ill patients compared to non-ICU patients [22].

The recurrence of AF after initially successful pharmacological or electrical conversion might be reduced by concurrent use of other drugs, for example, beta-blockers, but this must be investigated in future studies.

### 4.3. Safety

Vernakalant was well tolerated in our population of critically ill patients with a high prevalence of structural heart disease. Overall, the blood pressure values did not drop significantly 10, 30, 60, and 120 minutes after vernakalant infusion. However, 11 patients (31%) experienced a drop in blood pressure below 60 mmHg, which required the administration of additional vasopressors in 4 of them. The changes in blood pressure were only transient and no patient was harmed thereby. However, these results support close hemodynamic monitoring during the vernakalant infusion and the following 2 hours with the possibility to react in cases of  hypotension. The fact that blood pressure was significantly higher in nonresponders 30 and 120 minutes after the vernakalant infusion suggests that hypotension was not only a result of the study drug infusion but also a consequence of changes in heart rhythm and rate.

Kowey reported any episode of hypotension or bradycardia in 9.3% and 13.1% of vernakalant-treated cardiosurgical patients, respectively [17]. However, severe hypotension (systolic blood pressure <90 mmHg) and severe bradycardia (heart rate <40/min) occurred only in 5.6% and 3.7%, respectively. The ARVO study revealed severe adverse effects (symptomatic bradycardia or bradycardia <40/min, symptomatic hypotension of systolic blood pressure <85 mmHg, among others) during the 2-hour observation period in 5/116 (2.6%) of the patients [19]. The risk of hypotension might have been a bit higher in our ICU patients because of a lower blood pressure at baseline. Low blood pressure was easily manageable in the ICU setting but could potentially have led to more serious consequences without appropriate monitoring. Based on our data and the findings from the literature, we recommend to monitor blood pressure and heart rate during and for 120 minutes after the vernakalant infusion. No ventricular tachycardia or torsades de pointes occurred in the vernakalant-treated patients. Our results correspond to previous reports in which vernakalant did not prolong QTc or QRS intervals, and no proarrhythmic side effects were observed [14]. In the ACT trials I–IV, most common side effects were bradycardia and hypotension, but no deaths or proarrhythmic effects including torsades de pointes were reported in these studies [15–18].

### 4.4. Limitations of the Study

More detailed pieces of hemodynamic information including stroke volume or cardiac filling pressures were not available in our patients, as we only included hemodynamically stable patients in whom pulmonary artery catheters had been removed by the time of AF onset.

Vernakalant was also administered to intubated patients, in whom electrical cardioversion would have been a reasonable treatment option [23]. But as previously described, success rates after electrical conversion [21] might not be better than the ones published in our study. In nonintubated patients, drug treatment is favorable over electrical conversion, because it avoids the need for sedation with the subsequent risks of blood pressure compromise, respiratory arrest, and aspiration/asphyxia.

## 5. Conclusions

To our knowledge, this is the first report on the usefulness of vernakalant in ICU patients. We demonstrated that only a minority of postoperative ICU patients with new onset AF qualified for vernakalant when inclusion and exclusion criteria provided by the manufacturer were strictly applied. The conversion rate of >50% was comparable to published data in noncritically ill patients. Vernakalant administration was generally safe, but heart rate and blood pressure monitoring during and 2 hours after drug administration is still recommended because of the occasional episodes of hypotension and bradycardia. Due to its favorable safety profile, vernakalant represents an interesting treatment option for new onset AF in critically ill patients. Future research is needed to investigate if pre- and/or posttreatment with other antiarrhythmic drugs (e.g., with esmolol) can improve the conversion rate of vernakalant and reduce the risk of AF recurrence in responders.

## Figures and Tables

**Table 1:** Baseline characteristics.

Parameters	All (*n* = 32)	Responders (*n* = 17)	Nonresponders (*n* = 15)	*P* value
Age—years	74 (36–86)	74 (49–86)	76 (36–83)	0.77
Male gender	22 (69%)	10 (59%)	12 (80%)	0.27
Weight—kg	83 (58–108)	83 (62–100)	82 (58–108)	0.77
Height—cm	173 (145–184)	173 (150–180)	172 (145–184)	0.83
Type of surgery				
Coronary artery bypass	13 (41%)	7 (41%)	6 (40%)	1.00
Valve	18 (56%)	10 (59%)	8 (53%)	1.00
Major vascular	9 (28%)	4 (24%)	5 (33%)	0.70
SAPS	37 (18–64)	35 (18–64)	39 (21–63)	0.79
LV ejection fraction—%	57 (35–80)	60 (45–62)	55 (35–80)	0.59
Mechanical ventilation	14 (44%)	7 (41%)	7 (47%)	0.47
Dialysis	7 (22%)	3 (18%)	4 (27%)	0.86

LV: left-ventricular; SAPS: simplified acute physiology score. Results are given as median (minimum–maximum) or numbers (percentages). Groups were compared with Fisher's exact test or the Mann-Whitney *U* test, as appropriate.

**Table 2:** Laboratory values prior to treatment.

Parameters	All (*n* = 32)	Responders (*n* = 17)	Nonresponders (*n* = 15)	*P* value
Hematocrit—%	27 (20–38)	27 (20–38)	27 (24–38)	0.77
WBC (×10^9^/L)	12 (2.0–35)	12 (2.0–16)	12 (6.2–35)	0.60
Potassium—mmol/L	4.9 (4.0–5.2)	4.7 (4.3–5.2)	4.9 (4.0–5.1)	0.55
Magnesium—mmol/L	0.94 (0.77–2.3)	0.91 (0.77–1.6)	1.03 (0.88–2.3)	**0.03**
C-reactive protein—mg/L	123 (2–312)	122 (2–312)	124 (30–309)	0.79
Procalcitonin—ng/L	1.1 (0.30–29)	1.4 (0.50–29)	0.97 (0.30–17)	0.63
Creatine kinase—U/L	269 (11–762)	279 (11–762)	204 (39–567)	0.88
Troponin—ug/L	0.40 (0.04–4.8)	0.37 (0.04–4.8)	0.41 (0.07–1.5)	0.75
Creatinine—umol/L	92 (48–535)	92 (48–535)	92 (55–534)	0.88
Urea—mmol/L	11 (3.4–25)	8.8 (4.8–16)	11 (3.4–25)	0.18
Base excess	−0.55 (−6.6–+6.3	−0.55 (−2.7–+6.3)	−0.35 (−6.6–+1.5)	0.42
Bicarbonate—mmol/L	24 (20–30)	24 (22–30)	25 (20–26)	0.56

WBC: white blood cell count. Results are given as median (minimum–maximum). Groups were compared with the Mann-Whitney *U* test.

**Table 3:** Hemodynamic changes during treatment.

Parameters	All (*n* = 32)	Responders (*n* = 17)	Nonresponders (*n* = 15)	*P* value
Maximum HR—1/min				
At baseline	127 (70–163)	128 (75–160)	115 (70–163)	0.39
After 10 minutes	112 (60–146)	111 (70–140)	113 (60–146)	0.55
After 30 minutes	105 (61–155)	92 (64–146)	110 (61–155)	0.43
After 60 minutes	99 (69–139)	89 (70–130)	110 (69–139)	0.47
After 120 minutes	95 (70–158)	89 (70–146)	107 (74–158)	0.26
NA requirements				
At baseline	11 (34%)	6 (35%)	5 (33%)	1.00
Dose—mcg/kg/min	0.00 (0.00–0.10)	0.00 (0.00–0.05)	0.00 (0.00–0.10)	0.82
MAP				
At baseline	67 (54–90)	65 (54–86)	68 (62–90)	0.11
After 10 minutes	73 (57–103)	72 (57–92)	75 (62–103)	0.58
After 30 minutes	77 (55–103)	72 (55–88)	84 (59–103)	**0.02**
After 60 minutes	70 (54–101)	70 (54–101)	75 (55–96)	0.19
After 120 minutes	72 (60–106)	71 (60–85)	76 (62–106)	**0.04**
Lactate—mmol/L				
At baseline	1.1 (0.6–2.1)	1.0 (0.6–2.1)	1.1 (0.8–1.6)	0.92
After 120 minutes	1.2 (0.6–3.1)	1.1 (0.6–3.1)	1.3 (0.9–1.5)	0.79
SvO_2_—%				
At baseline	63 (49–75)	64 (51–75)	60 (49–75)	0.22
After 120 minutes	65 (48–75)	63 (48–75)	65 (55–72)	0.75

HR: heart rate; MAP: mean arterial pressure; NA: noradrenaline. Results are given as median (minimum–maximum) or numbers (percentages). Groups were compared with Fisher's exact test or the Mann-Whitney *U* test, as appropriate.

**Table 4:** Outcome.

Parameters	All (*n* = 32)	Responders (*n* = 17)	Nonresponders (*n* = 15)	*P* value
ICU length of stay—days	6 (1–62)	6 (1–23)	6 (2–62)	0.26
Sinus rhythm on ICU—discharge	23 (74%)	14 (88%)	9 (60%)	0.11
Survival	32 (100%)	17 (100%)	15 (100%)	1.00

Results are given as median (minimum–maximum) or numbers (percentages). Groups were compared with Fisher's exact test or the Mann-Whitney *U* test, as appropriate.
